# Effect of Exogenous Plant Debris and Microbial Agents on Phytoremediation of Copper-Contaminated Soil in Shanghai

**DOI:** 10.3390/plants11223056

**Published:** 2022-11-11

**Authors:** Qian Zhang, Hailan Fang, Kankan Shang

**Affiliations:** Shanghai Chenshan Botanical Garden, Shanghai 201062, China

**Keywords:** phytoextraction, plant composts, organic fertilizers, copper, orthogonal design

## Abstract

Bioaugmentation is an important measure for improving the efficiency of phytoremediation. The objective was to identify the role of exogenous plant debris with different processing and microbial agents for soil characteristics, copper bioavailability and phytoextraction. The experimental design consisted of four blocks, which were divided into 48 plots. Each plot was planted with *Fraxinus chinensis* and *Salix matsudana* × *alba*, which was added to plant composts, woody chips and effective microorganisms (EM) agents, using an orthogonal experimental design. The results showed that the order of bioaugmentation materials on the Cu phytoextraction of two woody species was plant composts > wood chips > EM agents. The best performance of *F. chinensis* was in the T15 treatment (30% plant composts + 7.5% wood chips + 40 mL·m^−2^), with phytoextraction of 33.66 mg·m^−2^, as well as 4.32 mg·m^−2^ in the T16 treatment (30% plant composts + 15% wood chips) of *S. matsudana* × *alba*. Cu was accumulated mainly in the roots of the two woody plants. The phytoextraction of the above-ground parts was promoted by bioaugmentation, due to the improvement in the physical soil characteristics and Cu bioavailability. The phytoextraction performance of *F. chinensis* was promoted by the improvement in the Cu concentration after treatments, while for *S. matsudana* × *alba*, it was the dry biomass. Thus, targeted strengthening measures should be to applied, to improve the efficiency of phytoremediation.

## 1. Introduction

Soil pollution is a great concern, as it poses threats to human beings in many industrial countries in the world. In China, copper concentration has exceeded the background values in almost all urban soils, ranging from 23.3 to 1226.3 mg·kg^−1^ [1,2]. It poses a major risk to plants, animals and humans, and has become an urgent health problem in urban ecosystems [3]. To solve this problem, more and more attention has been paid to the technical research of finding innovative solutions.

Phytoremediation, and more specifically phytoextraction, using fast-growing woody species is an efficient, nonharmful and potentially low-cost technique in comparison to traditional methods [4,5]. Phytoextraction has become a hot spot in the field of phytoremediation of woody plants, because of its well-developed roots, rapid growth and large biomass [6,7]. The efficiency of phytoextraction depends on the capacity of woody species to accumulate and transfer heavy metals in their above-ground tissues. The actual efficiency of phytoextraction depends on the establishment and survival of woody species on contaminated sites [8], as well as their convenient management techniques in the field. As woody species commonly used for ornamental purposes, *Fraxinus chinensis* and *Salix matsudana* × *alba* have good performances in soil phytoremediation, can quickly adapt to the urban environment, and are capable of enduring abiotic stresses [9].

Phytoextraction efficiency is also influenced by cultivation conditions and management measures in the field [10,11]. Bioaugmentation measures, such as fertilizer, organic-matter conditioner and microbial agents are intensive methods which are used for the enhancement of phytoextraction [12]. In general, plant debris is also used for soil remediation, common as a fertilizer [13,14]. However, the role of organic matter in the bioavailability and phytoremediation of heavy metals is still divergent [12]. On the one hand, organic matter can reduce the bioavailability of heavy metals in the soil by direct adsorption, chelation, and reduction conditions formed by decomposition [15], thus inhibiting the absorption and accumulation of heavy metals by plants [16]. On the other hand, most organic matter can promote desorption of heavy metals from the soil surface by releasing a large amount of water-soluble organic matter, and improve its bioavailability [17]. Most studies show that the water-soluble organic matter of manure, straw and sludge can inhibit the adsorption of heavy metals in soil, and improve its effectiveness and mobility [18]. It has also been found that organic matter such as plant composts, mushrooms, and food waste can improve the phytoextraction efficiency used for the micro-cuttings planting of *S. matsudana* × *alba* [19]. Moreover, microorganisms can also improve phytoremediation efficiency by changing the bioavailability of metals through secreted metal chelates [20], biosurfactants [21] and the redox reaction [22]. In addition, they can promote plant growth and survivability. Effective microorganisms (EM) can achieve the purpose of microbial remediation of soil polluted by low-concentration heavy metals [23], which are a mixed microbial group in an appropriate proportion composed of more than 80 species of microorganisms, including photosynthetic bacteria, lactic acid bacteria, actinomycetes and filamentous bacteria, etc. [24]. Meanwhile, the addition of EM agents to the soil can also improve the soil microbial-community structure [25] and the soil nutrients, by accelerating the decomposition and transformation of organic matter [26] and promoting plant photosynthesis and metabolism [27]. Therefore, exploring the influence of the mechanism of organic matter and microorganisms on phytoextraction is useful for improving the efficiency of fast-growing woody species.

The objective of this study is to explore the role of exogenous plant debris and microbial agents on the phytoextraction of Cu in a field trial, and to explain their accumulation mechanism using correlation analyses for soil characteristics, rhizosphere microbial diversity, and phytoremediation ability. The optimum strengthening measures for the remediation of Cu pollution by *F. chinensis* and *S. matsudana* × *alba* was explored through the application of plant debris-derived products and microbial agents. This will facilitate the rapid promotion of Cu phytoremediation in urban brownfield, as well as providing solutions for the recycling of organic waste.

## 2. Materials and Methods

### 2.1. Site and Stand Description

The experimental site was located in the grounds of the Chenshan Botanical Garden (31°4′39″ N, 121°11′12″ E) in Shanghai, China. Shanghai lies in the northern subtropical zone, and has a subtropical monsoon climate. Its average annual temperature is 17.8 °C, with an annual precipitation of 1457.9 mm, of which 60% is concentrated in the rainy season from May to September, and an average annual evaporation of 1257 mm [8]. The average humidity is 64.7%, and the average wind speed is 4.6 m·s^−1^. An appropriate amount of CuCl_2_ dry powder was used to spike the soil, and was mechanically incorporated into the first 25 to 30 cm of soil and left to equilibrate for four months, to act as simulated copper-contaminated soil. More details about the large experimental design will be shown; it was created based on a previous experiment in 2016 by Vincent et al. (2018) [8] and Shang et al. (2020) [28].

**Figure 1:**
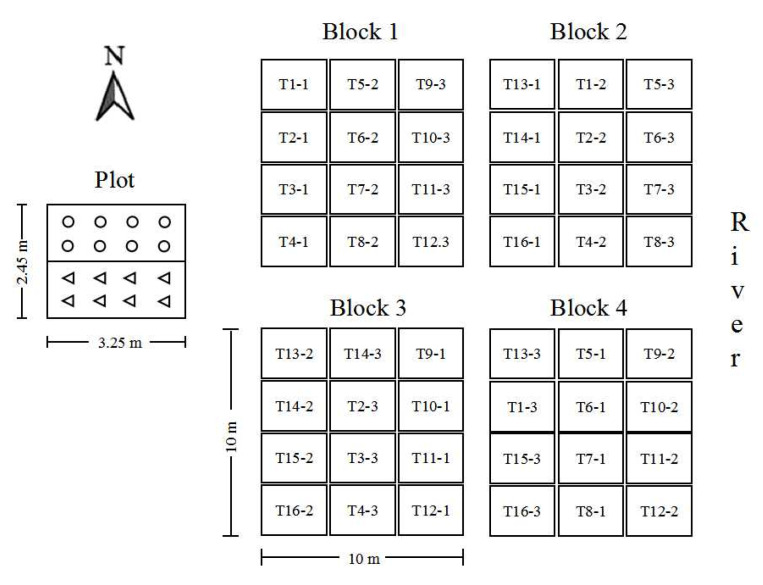
Experimental design of different bioaugmentation measures and planting modes. Note: the circle represents *F. chinensis*; the triangle represents *S. matsudana* × *alba*.

Four Cu-spiked uniform blocks (each 10 m × 10 m) were redesigned to carry out this study. The Cu concentration of block 1–4 were 652.8, 634.0, 639.7 and 619.3 mg·kg^−1^, respectively. Each block was randomly divided into 12 plots of 8 m^2^ (Figure 1). In each sub-plots plant composts, wood chips, and effective microorganisms (EM) agents were mixed into the 0–45 cm-deep soil on March 2021. Sixteen different treatments were set up using L16(4^3^) orthogonal experimental design (Table 1), and are illustrated in Figure 1. Wood chips and plant composts were derived from garden waste. The characteristics of the soils, plant composts and wood chips are presented in Table 2. The EM agents holding the number of active bacteria ≥ 10 billion·mL^−1^ were derived from Jiangxi Tianyi Biotechnology Co., Ltd. EM agents were diluted to 10 L with water, using different treatments, before being put in the soil.

The tested plants were *Salix matsudana* × *alba* and *Fraxinus chinensis*, with good performance in the previous test and which were ideal woody species for the phytoremediation of heavy metal Cu pollution in urban soil [9]. In each plot, eight *S. matsudana* × *alba* cuttings with a length of approximately 25 cm and eight one-year-old *F. chinensis* seedlings were planted on April 2021, with a total of 768 individuals. During the growing season, weeding was manually performed twice, in June and September. Irrigation was only carried out at the beginning of the experiment.

### 2.2. Soil Sampling and Analysis

To evaluate the soil characteristics after the materials addition, a mixed sample from three points per plot of 0–30 cm-soil layer were taken from soil samplers (φ5 cm) along the diagonal line, on June 2021. Soil samples were air-dried, crushed, sieved (0.149 mm mesh) and stored in sealed plastic bags [29]. Soil cores were also collected (using a 200 cm^3^ cylinder) from each plot, for determination of BD and maximum water-holding capacity [30]. SOM was determined by the potassium dichromate oxidation-heating method [31]. Hydrolysable nitrogen was determined by the alkaline-hydrolysis diffusion method [32]. Cation exchange capacity was determined by the ammonium chloride–ammonium acetate exchange method [33]. Soil pH and electrical conductivity were determined using a pH meter (Hach, Loveland, CO, USA) with a measuring range of −2.00 to 14.00, and an electrical conductivity instrument (Leici Company, Shanghai China) with a measuring range of 0.0 to 10.0 ec in the supernatant of 2:5 and 1:5 soil and water mixtures, respectively [34,35]. Water-soluble Cu was measured using inductively coupled plasma-mass spectrometry (ICP-MS) on the supernatant of 1:12.5 soil-to-water ratio. The suspension reacted on a reciprocal shaker for 2 h, was centrifuged (1400× *g*) for 10 min, filtered with a nylon membrane (0.45 mm), and stored in 0.2% trace metal grade HNO_3_ [36].

### 2.3. Rhizosphere Soil Sampling and Microorganism Determination

Rhizosphere soil, i.e., the soil adhering to plant roots, was collected by cutting the fine roots of *F. chinensis* randomly from each plot in June 2021. Each sample was put in an aseptic bag and kept frozen until transportation to the laboratory. Each sample was transferred to sterile 50 mL centrifuge tubes containing 20 mL of sterile 10 mM PBS solution, placed in a full temperature shaker at 120 rpm·min^−1^, and shaken at room temperature for 20 min [37]. The root systems were removed from the 50 mL centrifuge tubes using sterile tweezers, and the remaining suspension was centrifuged at high speed (6000× *g*, 4 °C) for 20 min, to collect the rhizosphere soil for DNA extraction and 16S ribosomal RNA gene amplicon sequencing.

Genomic DNA was extracted using the OMEGA Soil DNA Kit (D5625-01). The quantity and quality of the extracted DNA was examined using an Eppendorf RS232G UV-Vis spectrophotometer. The V3-V4 region of 16S rRNA genes were PCR amplified from bacteria compatible with the primers 338F (ACTCCTACGGGAGGCAGCAG) and 806R (GGACTACHVGGGTWTCTAAT) [38]. The PCR amplification products were sequenced using illumina MiSeq at Shanghai Meiji Biomedical Technology Co., Ltd. These sequence data were filtered and analyzed using the quantitative analysis of microbial ecology (QIIME) [39]. OTU clustering was carried out for non-repetitive sequences employing a 97% similarity. Based on the silver database, the RDP classifier Bayesian algorithm (with a default confidence threshold of 0.7) was used to annotate the selected OTU representative sequences, and a subsequent sequencing-data analysis was carried out. The alpha diversity index of the rhizosphere microorganisms under different intensive treatments was counted; the Shannon index was used to reflect the microbial-community diversity, the Chao index was used to reflect the microbial-community richness, and the Shannoneven index was used to reflect the microbial-community uniformity. Boxplots were produced for the soil microbial diversity index, using the software R.

### 2.4. Plant Sampling and Cu Concentration Analysis

In order to remove the maximum contaminants, all plants were coppiced at the end of the growth season. To evaluate the yield, the above-ground biomass (shoots and leaves) of all plants was weighed in the field, using an electronic scale (Xiangshan Inc. model ACS- JC21D). Portions of the plants were oven-dried at 80 °C (to a constant mass) before being reweighed, to determine the average dry biomass. The below-ground biomass of all plants was the average root system estimated by the above-ground parts.

Dry plant tissues from each sample were ground with a stainless-steel grinder until all particles passed through a 0.149 mm nylon sieve, to determine the Cu concentration. Approximately 0.5 g of these samples were digested with a mixture of concentrated HNO_3_ (10 mL), 30% H_2_O_2_ (1 mL), and concentrated HCl (2 mL) in Teflon tubes. These digested solutions had a constant volume of 25 mL with deionized water, and 10 mL of the suspensions were filtered through a nylon membrane (Magna-0.45 mm). The Cu concentration of these digested solutions were analyzed, using ICP-MS (Agilent ICPMS 7700) [40]. The translocation factor refers to the efficiency of the plant in translocating the accumulated metal from its roots to the shoots [41].

### 2.5. Statistical Analysis

All collected data were analyzed using EXCEL 2010 and SPSS 22.0 software. These random factor bioaugmentation materials, as well as the plant species, were compared. The mixed-model ANOVA (Tukey HSD and T-test) was carried out, to contrast the levels of the independent variables, and differences were deemed significant when *p* < 0.05. The relationships among soil characteristics, growth, and Cu accumulation in the plant tissue were analyzed using the Pearson correlation coefficient. A comprehensive assessment of the most appropriate measures was determined through a range analysis. In the range analysis, Ki (i = 1, 2, 3, 4) represented the sum of measured values of each factor at the same level, which distinguished the worst level and the best level of each influencing factor. R represented the range between different levels of the same factor. The primary and secondary factors were measured by the magnitude of the range (R), which indicated that this factor had a greater impact on the test results. On the other hand, it indicated that the influence of this factor on the test was relatively small [42].

## 3. Results

### 3.1. Changes in Soil Characteristics

The soil characteristics were obviously changed by different bioaugmentation measures, as shown in Table 3. With the increase of plant compost, maximum water-holding capacity (MWHC), soil organic matter (SOM), hydrolysable nitrogen (HN) concentration and cation-exchange capacity (CEC), were increased. Meanwhile, the bulk density (BD) was decreased and the water-soluble copper (WS-Cu) concentration had a peak in the 15% plant composts. When the addition of plant composts was 30%, the BD of T13, T14, T15 and T16 were 0.77, 0.89, 0.84 and 0.84 g·cm^−3^, respectively, which was significantly lower than the non-compost treatments (*p* < 0.05). The MWHC of four treatments (T13, T14, T15 and T16) added to 30% plant composts was significantly higher than that of the other treatments, and the maximum value was 814.32 g·kg^−1^ in T13. The SOM, HN concentration and CEC of the treatment group with 30% plant composts (T13-T16) were highest, with average values of 36.19 g·kg^−1^, 141.19 mg·kg^−1^ and 17.31 cmol(+)·kg^−1^, respectively. There was no obvious regularity or significant difference in pH and electrical conductivity (EC) among the different bioaugmentation measures. The soil WS-Cu concentration in the T9 and T11 treatments were 2.58 and 2.56 mg·kg^−1^, respectively, which was significantly higher than that of T1.

### 3.2. Microbial Diversity in Rhizosphere Soil

**Figure 2:**
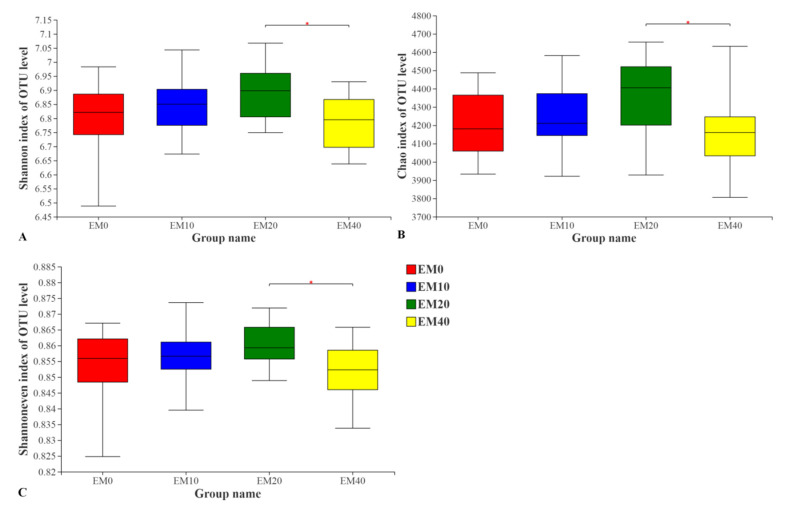
Alpha diversity index statistics of rhizosphere microorganism of *F. chinensis* in different treatments. (**A**) Shannon index of OTU level; (**B**) Chao index of OTU level; (**C**) Shannoneven index of OTU level. Note: *p* < 0.05 is marked as asterisk.

A total of 2,902,193 sequences were obtained from all the 48 samples after removal of the index and primer sequences, quality-control filter and rarefaction. The average length of the sequence was 418 bp, which was consistent with the length of the 16S rDNA V3 + V4 region. The data of 48 samples were flattened, and each sample contained 48,366 effective sequences. In line with the OTU, taxon with 16S rDNA similarity ≥ 97%, 8307 OTUs were obtained after clustering, and 46 phyla, 155 classes, 364 orders, 592 families, 1119 genera and 2367 species were identified. According to the microbial diversity indexes of the different treatments, microbial agents were the primary factor affecting rhizosphere microbial diversity, so they were grouped according to the dosage of effective microorganism (EM) agents. This showed that the value of Shannon, Chao and Shannoneven was EM20 > EM10 > EM0 > EM40, respectively, through the analysis of the differences between the index groups (Figure 2). The three indexes in the treatment with 20 mL·m^−2^ EM agents were significantly different from those in the treatment with 40 mL·m^−2^ EM agents (*p* < 0.05).

### 3.3. Cu Concentration in Plant Tissues

**Figure 3:**
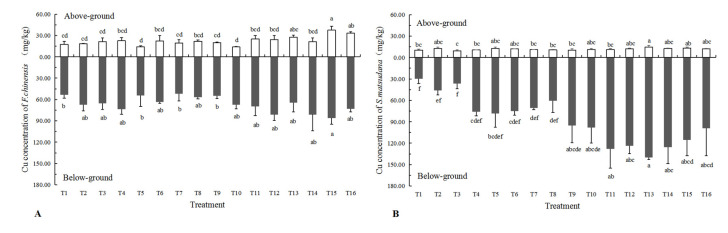
Cu concentrations of above-and below-ground parts of two woody species. (**A**) Cu concentration of *F. chinensis*; (**B**) Cu concentration of *S. matsudana* × *alba*. Note: Values are mean ± SE. Different letters in a row indicate a significant difference, *p* < 0.05.

In general, the Cu concentration in the below-ground parts of plants was significantly higher than that in above-ground parts, especially in the tissues of *S. matsudana* × *alba* (Figure 3). With the increase in the amount of plant composts added, the Cu concentration in the plant tissues of two woody species showed an upward trend, and 30% plant composts treatments had a significantly higher concentration than that of the others. As shown in Figure 3A, the Cu concentration of the above-ground parts of *F. chinensis* in the T15, T16 and T13 treatments were 37.55, 33.45 and 27.62 mg·kg^−1^, respectively. The highest concentration of below-ground parts was in T15, which was 85.88 mg·kg^−1^. As shown in Figure 3B, the Cu concentration of the above-ground parts of *S. matsudana* × *alba* in the T13, T15 and T14 treatments were 14.67, 13.16 and 12.82 mg·kg^−1^, respectively. The highest concentration of below-ground parts was in T13, which was 139.83 mg·kg^−1^.

The addition of plant composts had a significant effect on the translocation factor of plants, showing different trends in the two tree species. The translocation factor of *F. chinensis* with 30% plant composts treatments was higher than that of other treatments, and the highest treatment was T16, T15 and T13, which were 0.46, 0.44 and 0.43, respectively. The transfer coefficient of *S. matsudana* × *alba* decreased significantly when adding plant composts, because the Cu concentration of below-ground parts increased greatly.

### 3.4. Yield and Cu Quantity Extracted by the Plants

**Figure 4:**
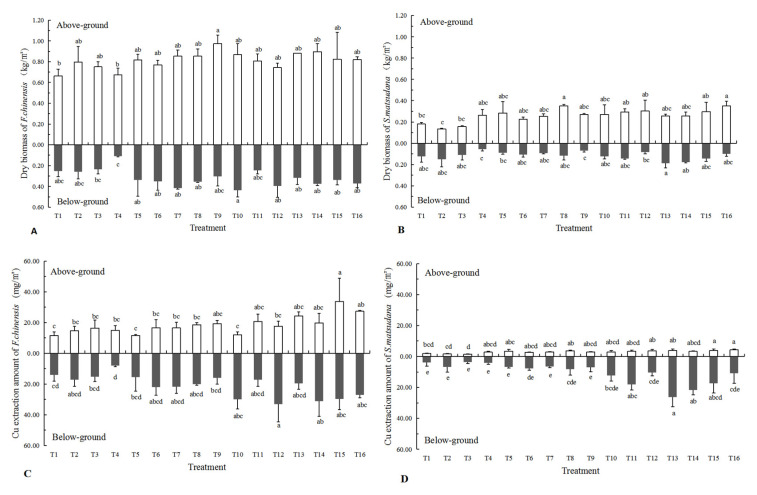
Yields and Cu extraction of above-and below-ground parts of two woody species. (**A**) Dry biomass of *F. chinensis*; (**B**) Dry biomass of *S. matsudana* × *alba*; (**C**) Cu extraction amount of *F. chinensis*; (**D**) Cu extraction amount of *S. matsudana* × *alba*. Note: Values are mean ± SE. Different letters in a row indicate a significant difference, *p* < 0.05.

The yield was evaluated at the end of the growing seasons (Figure 4). The biomass and Cu quantity extraction of the above-ground, below-ground and whole plant of two woody species had improved to a certain extent, among different bioaugmentation measures. The performance of *F. chinensis* was better than *S. matsudana* × *alba*, especially in 30% plant composts treatments.

The maximum biomass of above-ground parts of *F. chinensis* was in T9, with 0.97 kg·m^−2^, with 0.44 kg·m^−2^ of below-ground parts in T10. The biomass of *F. chinensis* treated with 7.5%, 15% and 30% plant composts was higher than that of treatments without plant composts (Figure 4A). Meanwhile, the Cu extraction of the above-ground parts of *F. chinensis* in T15 was the highest, reaching 33.66 mg·m^−2^, and the differences between the treatments without plant composts and with 7.5% plant composts, were significant. The same trend was indicated in the below-ground parts, except for that of T12 (Figure 4C). Similarly, the maximum biomass of the above-ground parts of *S. matsudana* × *alba* was in T8 and T16, which reached 0.35 kg·m^−2^, while the maximum biomass of below-ground parts was 0.19 kg·m^−2^ in T13 (Figure 4B). With the increase in the plant composts addition, the biomass of *S. matsudana* × *alba* also increased gradually, and reached the maximum value in the 30% plant composts addition. Meanwhile, higher Cu extractions of above-ground parts were in T15 and T16, with 3.86 and 4.32 mg·m^−2^, respectively. With the increase in plant composts addition, the Cu extraction of below-ground parts increased significantly, and reached a peak value of 26.23 mg·m^−2^ in T13 (Figure 4D).

### 3.5. Correlation Analysis

The correlation analysis results of the above-ground indexes and soil characteristics are shown in Table 4. Although there were some differences in the influence of soil on Cu concentration and dry biomass of above-ground parts between the two woody plants, the influence of Cu phytoextraction was consistent. The Cu phytoextraction of the two woody plants was significantly negatively correlated with BD (R^2^ = −0.729 and −0.706), and significantly positively correlated with MWHC (R^2^ = 0.720 and 0.692) and Ws-Cu (R^2^ = 0.736 and 0.647).

There were differences in the phytoextraction performance of the above-ground parts between F. chinensis and S. matsudanas × alba. The Cu extraction of the above-ground parts of F. chinensis was significantly positively correlated with the Cu concentration in the plant tissue (R = 0.949), while S. matsudana × alba mainly depended on dry biomass (R = 0.921) (Table 5).

### 3.6. Comprehensive Assessment by Range Analysis

The results of a range analysis on the Cu extraction of above-ground parts are shown in Table 6. The most important factor for both *F. chinensis* and *S. matsudana* × *alba* was the plant composts, identified by range value. The grade of the three factors was the following: plant composts > wood chips > EM agents. The optimum ratio of Cu extraction of the above-ground parts in *F. chinensis* was 30% plant composts + 7.5% wood chips + 40 mL·m^−2^ EM agents, while the above-ground parts of *S. matsudana* × *alba* was 30% plant composts + 15% wood chips+ 40 mL·m^−2^ EM agents.

## 4. Discussion

### 4.1. Role of Exogenous Plant Debris and Microbial Agents on Phytoremediation

This study aimed to improve the copper phytoextraction of plant tissues by means of the enhancement of physical, chemical and biological soil characteristics, using plant composts, wood chips, and effective microorganisms (EM) agents. The order of these factors in the enhancement of Cu extraction in the above-ground parts of *F. chinensis* and *S. matsudana* × *alba* was plant composts > wood chips > EM agents. The results of the correlation analysis showed that the phytoextraction amount was improved by the enhancement of physical soil characteristics and the metal bioavailability in *F. chinensis* and *S. matsudana* × *alba*.

As a raw material, plant debris is produced in bark blocks, wood chips, composts, earthworm manure and biochar, to improve urban soil. Generally considered as a physical-structure conditioner, wood chip is widely used as organic mulch, but rarely used for raw-materials mixture. Compost can effectively improve soil characteristics, especially fertility [43,44], through organic matter, nitrogen and phosphorus in the soil [45]. And that compost can provide soil microorganisms with nutrients needed for reproduction, and enhance microbial activity [46]. At the same time, microorganisms can promote the decomposition of organic matter in compost, effectively control nitrogen loss, and play a role in nitrogen fixation and fertilizer conservation [47]. Under the synergetic additions of three bioaugmentation materials, bulk density (BD), maximum water-holding capacity (MWHC), soil organic matter, hydrolysable nitrogen, and cation-exchange capacity changed significantly among the different treatments, especially with plant composts increased. Unexpectedly, plant composts had a greater impact on BD and MWHC than wood chips, due to the large difference in proportion.

The bioavailability of heavy metals directly affects the phytoextraction efficiency of plants, and its effect even exceeds the total concentration of heavy metals [48]. Organic matters such as compost, manure and feedstock are generally used to promote phytoremediation in contaminated soil, as well as for improvement in properties and quality [14,19]. There are differences between the role of heavy-metal bioavailability and phytoextraction efficiency. On one hand, such organic matters containing rich humus can significantly improve the biomass of plants and the bioavailability of heavy metals in the soil, by releasing a large amount of water-soluble organic matter [13,17]. Microorganisms will produce a variety of amino acids and low-molecular-weight organic acids during their growth and metabolism, which can also activate solid heavy metals in the soil [49]. On the other hand, organic matter can also reduce the bioavailability of heavy metals in the soil through direct adsorption, chelating and the reduction conditions formed by decomposition [15], and inhibit the absorption and accumulation of heavy metals by plants [16,17]. A certain concentration of EM agents can effectively improve the diversity, richness and uniformity of *F. chinensis*. A high concentration of microbial agents may lead to excessive microbial density in the soil, and inhibit the physiological activities of microorganisms. When more than 15% plant compost was used in this experiment, the water-soluble Cu (WS-Cu) concentration reached a peak, due to the absorption of plant composts, which may be greater than the desorption. It was reported that the form of heavy metal in the soil was related to the pH of the soil, and a low pH value can reduce the adsorption rate of heavy metal, and increase the content of available metal [50]. However, no significant correlation between WS-Cu concentration and pH was found in this study, which may be due to the alkaline soil characteristics in this experiment.

The EM agent was the primary factor affecting the microbial diversity index. In the plot where *F. chinensis* was planted, the diversity, richness and uniformity of soil microorganisms were improved by adding a certain concentration of EM agents. EM agents can improve soil microbial-community structure, increase soil microbial quantity and soil enzyme activity. A high concentration of EM agents may lead to excessive microbial density in the soil, and inhibit the physiological activities of microorganisms [51]. It was found that microorganisms can promote plant growth [52,53] and improve the survivability of heavy metal [54,55] through various mechanisms. The same result was found in this experiment: the dry biomass of *F. chinensis* was significantly positively correlated with the microbial diversity indexes. However, there was no significant relationship between the microorganism indexes and phytoextraction ability. This may be attributed to the insignificant correlation between microbial diversity indexes and Cu concentration.

### 4.2. Phytoextraction Performance of F. chinensis and S. matsudanas × alba

A high accumulation of metals in roots and low transport of heavy metals to the shoots were reported to be the key mechanisms in protecting plant organs involved in photosynthesis [56]. High phytoremediation ability can be found in fast-growing woody species, mainly fixed in the roots [28]. The Cu concentration of the below-ground parts of two woody species was higher than that in the above-ground tissues, even after bioaugmentation measures, especially in the tissues of *S. matsudanas* × *alba*. This is due to the low Cu mobility in the soil [57]. Most of the heavy-metals tests showed that the highest accumulation was recorded in the roots of willow, followed by the leaves and stems, which supported our current results [58].

According to the Cu concentration in the above-ground and below-ground parts, the transfer ability of *F. chinensis* was stronger than that of *S. matsudana* × *alba*. Cu is accumulated and stored in roots, and then transported to the above-ground parts, which is a way for the plant to resist heavy metal [59]. When stress occurs, roots can also make an adaptive response, by influencing the behavior of the above-ground parts through the transmission of information materials [56]. It was reported that the root activity of *F. chinensis* was significantly higher than that of *S. matsudana* under high Cu-concentration stress (>500 mg·kg^−1^) [60]. Therefore, it can be concluded that *S. matsudana* × *alba* has a stronger ability to fix Cu in the roots system, and *F. chinensis* has a stronger potential to extract Cu.

In general, the phytoremediation potential in the former research showed different patterns that were dependent on the heavy metal type and tree genotype [61]. The phytoextraction performance of *F*. *chinensis* was promoted by the increase in Cu concentration in the plant tissue under bioaugmentation, while *for S. matsudana × alba,* it was the dry biomass. The phytoremediation mode of the two woody plants was different, which may be due to the difference of the translocation factor.

## 5. Conclusions

The phytoextraction of two woody plants was promoted by exogenous plant debris and microbial agents, due to the improvement in physical soil characteristics and copper bioavailability. Plant composts play the best role for soil improvement and copper phytoextraction in comparison with woody chips and EM agents. The best performance of *F. chinensis* was in the T15 treatment (30% plant composts + 7.5% wood chips + 40 mL·m^−2^) with phytoextraction of 33.66 mg·m^−2^, as well as 4.32 mg·m^−2^ in the T16 treatment (30% plant composts + 15% wood chips) of *S. matsudana* × *alba*. The Cu accumulation in the above-ground parts of *F. chinensis* was attributed to the Cu concentration of plant tissues, while biomass was the primary determinant of *S. matsudana* × *alba*. Thus, according to the promoting effect of different tree species, we need to choose targeted strengthening measures to improve the efficiency of phytoremediation.

## Figures and Tables

**Table 1:** Treatments of orthogonal array test.

Treatments	Factors		
	Plant Composts (%vol)	Wood Chips (%vol)	EM Agents (mL·m^−2^)
T1	0	0	0
T2	0	3.75	40
T3	0	7.5	20
T4	0	15	10
T5	7.5	0	40
T6	7.5	3.75	0
T7	7.5	7.5	10
T8	7.5	15	20
T9	15	0	20
T10	15	3.75	10
T11	15	7.5	0
T12	15	15	40
T13	30	0	10
T14	30	3.75	20
T15	30	7.5	40
T16	30	15	0

**Table 2:** Characteristics of the soils, plant composts and wood chips.

Parameters	Soils	Plant Composts	Wood Chips
PS (cm)	/	0.3–1.0	2.0–3.0
BD (g·cm^−3^)	1.31	0.40	0.38
pH	8.20	7.57	7.85
SOM (g·kg^−1^)	24.2	69.89	109.20
TN (g·kg^−1^)	2.36	2.37	2.45
TP (g·kg^−1^)	1.62	1.74	1.75

Note: PS, BD, SOM, TN and TP are the abbreviations for particle size, bulk density, soil organic matter, total nitrogen, and total phosphorus, respectively.

**Table 3:** Soil physiochemical characteristics in different treatments.

Treatments	BD (g·cm^−3^)	MWHC (g·kg^−1^)	SOM (g·kg^−1^)	HN (mg·kg^−1^)	CEC (cmol(+)·kg^−1^)	EC (uS·cm^−1^)	pH	WS-Cu (mg·kg^−1^)
T1	1.21 ± 0.07 a	437.40 ± 28.68 f	24.54 ± 2.60 c	111.53 ± 16.52 cd	12.48 ± 0.02 f	140.57 ± 12.14 b	8.08 ± 0.04 abcd	1.38 ± 0.07 bcd
T2	1.05 ± 0.07 abc	528.14 ± 53.95 def	33.24 ± 7.18 bc	109.79 ± 23.04 cd	11.98 ± 0.33 f	149.60 ± 7.61 ab	8.09 ± 0.05 abcd	1.79 ± 0.08 abcd
T3	1.06 ± 0.09 abc	497.35 ± 59.23 ef	31.83 ± 4.79 bc	124.79 ± 20.55 bcd	14.72 ± 1.20 cdef	153.57 ± 7.83 ab	8.05 ± 0.07 abcd	1.96 ± 0.44 abcd
T4	1.05 ± 0.04 abc	524.69 ± 39.71 def	30.02 ± 5.82 bc	107.21 ± 30.57 cd	14.29 ± 1.56 def	155.70 ± 7.75 ab	8.12 ± 0.10 abcd	1.29 ± 0.51 cd
T5	1.13 ± 0.00 ab	489.68 ± 3.17 ef	31.50 ± 3.02 bc	117.01 ± 13.58 bcd	16.12 ± 0.88 abcde	154.73 ± 8.86 ab	7.94 ± 0.13 cd	1.50 ± 0.07 abcd
T6	1.02 ± 0.11 abcd	548.64 ± 70.62 cdef	27.95 ± 2.63 bc	100.38 ± 11.95 d	14.43 ± 0.54 def	139.43 ± 6.99 b	8.15 ± 0.02 ab	1.50 ± 0.63 abcd
T7	0.92 ± 0.06 cde	630.13 ± 50.67 bcde	38.48 ± 3.43 ab	134.45 ± 8.31 bcd	15.56 ± 0.10 abcde	155.03 ± 5.41 ab	8.20 ± 0.02 a	2.50 ± 0.14 ab
T8	1.12 ± 0.04 ab	481.39 ± 37.33 ef	33.16 ± 2.16 bc	127.72 ± 15.07 bcd	15.58 ± 1.46 abcde	153.63 ± 8.23 ab	8.13 ± 0.02 abc	2.12 ± 0.45 abcd
T9	1.05 ± 0.03 abc	531.46 ± 19.81 def	45.71 ± 1.11 a	192.89 ± 14.62 a	17.48 ± 0.22 abc	167.00 ± 10.48 a	7.93 ± 0.07 d	2.58 ± 0.76 a
T10	0.95 ± 0.04 bcde	615.15 ± 25.45 bcde	33.39 ± 0.97 bc	119.32 ± 3.27 bcd	13.65 ± 0.50 ef	152.90 ± 7.54 ab	8.08 ± 0.07 abcd	2.46 ± 0.30 abc
T11	0.98 ± 0.12 bcd	588.66 ± 93.14 bcdef	29.63 ± 2.04 bc	114.19 ± 12.96 bcd	16.15 ± 0.07 abcde	152.60 ± 4.48 ab	8.15 ± 0.07 ab	2.56 ± 0.11 a
T12	1.08 ± 0.10 abc	504.64 ± 71.29 ef	29.04 ± 6.89 bc	113.37 ± 23.85 bcd	15.47 ± 1.13 bcde	149.07 ± 12.71 ab	8.17 ± 0.07 ab	2.00 ± 0.36 abcd
T13	0.77 ± 0.07 e	814.32 ± 78.91 a	36.00 ± 2.85 abc	132.13 ± 9.54 bcd	15.63 ± 0.69 abcde	155.77 ± 8.94 ab	8.04 ± 0.03 abcd	1.42 ± 0.15 abcd
T14	0.89 ± 0.10 cde	688.07 ± 87.69 abcd	38.48 ± 4.53 ab	154.42 ± 24.39 abc	17.09 ± 1.32 abcd	159.30 ± 8.80 ab	8.00 ± 0.03 bcd	1.81 ± 0.32 abcd
T15	0.84 ± 0.06 de	709.82 ± 85.97 abc	31.25 ± 2.41 bc	115.36 ± 10.50 bcd	18.07 ± 2.34 ab	148.93 ± 5.39 ab	8.05 ± 0.05 abcd	1.25 ± 0.14 d
T16	0.84 ± 0.02 de	741.86 ± 31.75 ab	39.01 ± 5.02 ab	162.86 ± 13.84 ab	18.46 ± 0.57 a	161.93 ± 4.97 ab	7.99 ± 0.05 bcd	1.50 ± 0.07 abcd

Note: Values are mean ± SE. Different letters in a row indicate a significant difference, *p* < 0.05.

**Table 4:** Correlation analysis between above-ground indexes and soil characteristics.

Index	Cu Concentration		Dry Biomass		Cu Phytoextraction	
	*F. chinensis*	*S. Matsudanas* × *alba*	*F. chinensis*	*S. Matsudanas* × *alba*	*F. chinensis*	*S. Matsudanas* × *alba*
BD	−0.625 **	−0.596 *	−0.480	−0.309	−0.729 **	−0.706 **
MWHC	0.617 *	0.662 **	0.473	0.339	0.720 **	0.692 **
SOM	0.072	0.080	0.832 **	0.252	0.295	0.259
HN	0.115	−0.059	0.710 **	0.305	0.301	0.248
CEC	0.622 *	0.229	0.651 **	0.392	0.251	0.135
EC	0.088	−0.036	−0.440	−0.071	−0.162	−0.046
pH	−0.007	−0.193	0.503 *	0.095	−0.227	−0.047
WS-Cu	−0.374	−0.438	0.527 *	0.727 **	0.736 **	0.647 **
Shannon	0.079	/	0.617 *	/	0.216	/
Chao	0.079	/	0.612 *	/	0.238	/
Shannoneven	0.128	/	0.563 *	/	0.241	/

Note: ** Correlation is significant on 0.01 layer (double tail), * correlation is significant on 0.05 layer (double tail).

**Table 5:** Correlation analysis between Cu-extraction amount with Cu concentration and dry biomass of above-ground parts of *F. chinensis* and *S. matsudana* × *alba*.

Index	Cu-extraction Amount	
	*F. chinensis*	*S. matsudana* × *alba*
Cu concentration	0.949 **	0.520 *
Dry biomass	0.308	0.921 **

Note: ** Correlation is significant on 0.01 layer (double tail), * correlation is significant on 0.05 layer (double tail).

**Table 6:** Range analysis of Cu extraction of above-ground parts of two woody species.

Species		Factors		
		Plant Composts	Wood Chips	EM Agents
*F. chinensis*	K1	57.54	66.53	76.01
	K2	63.05	63.23	67.80
	K3	69.54	87.12	74.01
	K4	105.06	78.31	77.37
	R value	47.52	23.89	9.57
	Optimal level	30%	7.5%	40 mL·m^−2^
*S. matsudana* × *alba*	K1	7.88	12.07	12.32
	K2	12.75	10.66	12.36
	K3	12.73	11.47	11.32
	K4	15.29	14.45	12.65
	R value	7.41	3.79	1.33
	Optimal level	30%	15%	40 mL·m^−2^

## Data Availability

The data presented in this study are available on request from the corresponding author. The data are not publicly available, due to the restriction policy of the co-authors’ affiliations.
